# Sexual Activities as Risk Factors of Rotator Cuff Lesions: A Prospective Cohort Study

**DOI:** 10.1007/s11195-018-9543-y

**Published:** 2018-10-30

**Authors:** Alexandre Lädermann, Grégory Cunningham, Sylvain Chagué, Caecilia Charbonnier

**Affiliations:** 1 Division of Orthopaedics and Trauma Surgery, La Tour Hospital, Avenue J.-D. Maillard 3, 1217 Meyrin, Switzerland; 2 Faculty of Medicine, University of Geneva, Geneva, Switzerland; 3 Division of Orthopaedics and Trauma Surgery, Department of Surgery, Geneva University Hospitals, Geneva, Switzerland; 4 Division of Orthopaedics and Trauma Surgery, Hirslanden La Colline, Geneva, Switzerland; 5 Medical Research Department, Artanim Foundation, Geneva, Switzerland

**Keywords:** Shoulder pathology, Rotator cuff tear, Tendinopathy, Subacromial impingement, Sexual activity, Kinematics, Biomechanics, 3D simulation, Switzerland

## Abstract

The aim of this study was to analyze the occurrence of rotator cuff impingement due to reduction of subacromial space height during complex shoulder motion to determine safety of sexual activities. The hypothesis was that such activities could be deleterious if not performed with precaution. To use a patient-specific 3D measurement technique coupling medical imaging and optical motion capture to evaluate the safety of various sexual positions according to subacromial compression. Descriptive laboratory study. A volunteer couple underwent Magnetic Resonance Imaging (MRI) and motion capture of their shoulders. Five common active and passive sexual positions were evaluated. Significant differences in subacromial space height were observed between the different performed actions. All active sexual positions requiring important pressure on the hands or elbows (e.g., scorpio) or weight lifting (e.g., superman) caused subacromial impingement. No subacromial impingement was however observed during passive sexual activities (e.g., basset hound). This study indicates that some sexual positions could potentially place the rotator cuff at risk. Such high-tech investigation shows promise in the areas of cause, intervention and education. The present findings may assist health professionals in providing them with preventive measures and is highly relevant for decision-making regarding health promoting initiatives.

## Introduction

Tears of the rotator cuff are a frequent and a well-known cause of chronic pain and dysfunction in the shoulder [1]. Their aetiologies in non-traumatic situations are controversial, with two main theories described: an extrinsic mechanism, also called subacromial impingement, where symptoms are caused by compression of the rotator cuff [2], and an intrinsic one, where symptoms are thought to result from overload on degenerating rotator cuff tendons [3]. The authors of the present study have noted other alarming risk factors in recent publications that may impede with general quality of life. Indeed, it has been proved that almost all enjoyable activities (i.e., sports [4], good food associated to hypercholesterolemia [5], alcohol [6] or tobacco [7]) are deleterious for the rotator cuff.

Sexual activity is another known factor of emotional happiness [8]. However, no such association has been established with shoulder injuries yet. To rule out a ‘worst case scenario’, the authors investigated if the latter activity was also associated with rotator cuff overload in order to promote, if required, adequate injury prevention. This issue remaining rarely discussed and very limited objective data being available to propose recommendations, they felt the necessity to fill this gap. Simulating dynamically subacromial impingements during activities of daily living is challenging. This requires accurate 3D reconstruction of the shoulder bones, kinematics estimation of the joint during complex shoulder motion and evaluation of impingement using the subject-specific 3D models and kinematics. Fortunately, the authors previously developed and validated in different studies all the necessary tools to perform such challenging simulation [4, 9–11].

The aim of this study was to analyze the occurrence of rotator cuff impingement during complex shoulder motion using an in vivo technique combining optical motion capture and magnetic resonance imaging (MRI) to determine safety sexual activities for men. The hypothesis was that such activities could be deleterious for shoulders if not performed with precaution.

## Methods

### Subjects

This study was a prospective trial carried out on two healthy right-handed volunteers (one female, one male) that did not report previous shoulder injury or surgery. The age, weight, height and body mass index of the two subjects were 39 and 31 years, 161 and 180 cm, 52 and 80 kg, and 22.5 and 24.7 kg/m2, respectively. The dominant arm was used throughout testing. Young healthy subjects were chosen for two reasons: (1) patients presenting symptomatic rotator cuff tears are becoming younger [1] and are therefore sexually more active; (2) certain sexual positions tested being at risk or physically demanding, inclusion of healthy subjects was hence preferable to avoid any incident during motion capture. Exclusion criteria included previous pathology or surgery of the shoulders, any psychiatric condition such as obsessive–compulsive disorder (OCD, DSM-IV Code 300.3) or psycho-physiological sexual dysfunction. The latter was based on medical history collection, only. The study was approved by our hospital’s institutional review board (AMG-12.18).

### Study Variables

The outcome of interest was the impact of common sexual positions on subacromial space height.

## 3D Reconstruction, Kinematic Recording and Modelling

The volunteers underwent an MRI performed with a 1.5 T HDxT system (General Electric Healthcare, Milwaukee WI, USA). A dedicated shoulder surface coil was used. Three 3D MRI volumes were acquired: a cosmic 3D fast gradient echo sequence with fat saturation (section thickness 1.8 mm; no gaps; TR/TE ms 6.1/3.0; flip angle 45°) capturing from the acromion to approximately the mid-part of the scapula, a cosmic 3D fast gradient echo sequence (section thickness 4 mm; no gaps; TR/TE ms 5.7/2.8) capturing from the acromion to approximately the mid-shaft of the humerus, and a lava 3D fast gradient echo sequence (section thickness 5.2 mm; no gaps; TR/TE ms 3.7/1.7) capturing from the acromion to the elbow.

The MR images were manually segmented and a virtual 3D model of the shoulder complex was reconstructed using Mimics software (Materialize NV, Leuven, Belgium). For each volunteer, patient-specific 3D models of the shoulder bones (humerus, scapula, clavicle and sternum) were thus obtained.

The next step was motion recording. The two volunteers were equipped with a dedicated shoulder markers protocol [9], including 69 spherical retroreflective markers placed directly onto the skin using double sided adhesive tape.

**Fig. 1:**
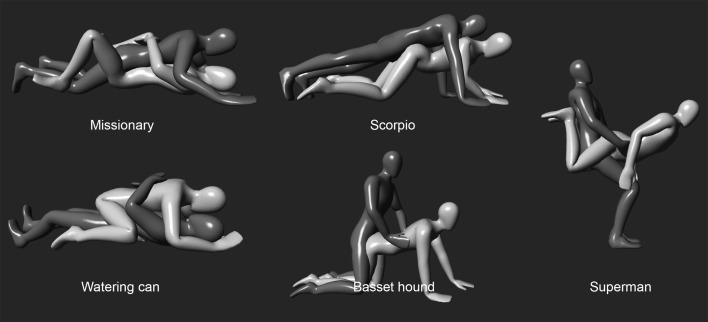
The five common sexual positions used in this study. In all images, the woman is represented in yellow and the man in green (Color figure online)

Then, after appropriate preliminaries, the two volunteers were asked to perform five common sexual positions (Fig. 1) divided into the following categories: “man-on-top” such as missionary and scorpio, “woman-on-top” such as watering can, “doggy style” such as basset hound and “standing” such as superman. A mattress was utilized when needed.

The female and male volunteers were asked to simulate each sexual position three times. Motion was recorded using a Vicon MXT40S motion capture system (Vicon, Oxford Metrics, UK) consisting of 24 cameras sampling at 120 Hz.

**Fig. 2:**
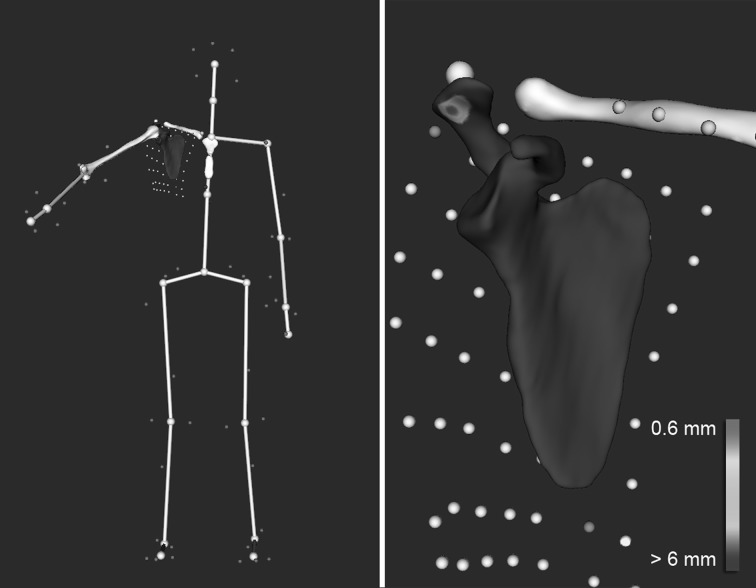
Visualization of the acromio-humeral distance during the 60° shopping angle. Left: joint pose computed by motion capture. Right: zoom in the shoulder. The colors represent the variations of distance between the acromion and humeral head. Red denotes the zone of minimum distance. Note: the humerus is not shown for clarity (Color figure online)

Shoulder kinematics were computed from the recorded markers’ trajectories using a validated biomechanical model [9] which accounted for skin motion artifacts. The model was based on a patient-specific kinematic chain using the 3D models reconstructed from participants’ MRI scan data and a global optimization algorithm with loose constraints on joint translations (accuracy: translational error < 3 mm, rotational error < 4°). As a result, the motion of the subject’s shoulder 3D models could be visualized at each point of the movement (see Fig. 2).

### Evaluation of Subacromial Space Height

Subacromial space height was assessed for all tested positions during sexual activity by measuring the minimum distance between the inferior acromial surface and the humeral head surface [10]. This distance was calculated in 3D based on the simulated bones models positions and was reported in millimeters. A color scale was used to map the variations of distance on the scapula surface, with red denoting the zone of minimum distance and other colors denoting the areas of increased distance (Fig. 2). Given the thickness of the potential impinged tissues, subacromial impingement was considered when the computed acromio-humeral distance was < 6 mm, as suggested in previous studies [12, 13].

### Statistical Analysis

For each shopping angle and sexual position, we calculated the minimal subacromial space height for the three trials performed. The mean and standard deviation (SD) were then computed for each positions.

## Results

Results are summarized in Table 1. No shoulder pathologies were noticed on MRI. Minimal subacromial height ranged between 0.91 mm and 6.2 mm for the sexual positions. The least favorable sexual activity with respect to subacromial space height was during the practice of the scorpio position.

**Table 1:** Minimal subacromial space height (mm) for each position (n = 3)

Man’s sexual positions	Mean	SD
Missionary	1.27	0.03
Scorpio	0.91	1.06
Watering can	6.1	0.22
Basset hound	6.2	0.17
Superman	1.76	1.60

Data are reported for the male participant performing three trials for each position

## Discussion

Subacromial impingement and superior rotator cuff pathologies are associated with dysfunction and pain related to the upper extremity [14]. Physicians and probably patients wonder about the risk related to sexual activities. Presently, surgeons or other healthcare professionals lack scientifically validated information on appropriate answers or guidelines and are therefore unable to provide specific instructions to patient’s inquiries. The hypothesis of this study was confirmed as it demonstrated that certain positions during sexual activities might participate to the development of rotator cuff degeneration. The study revealed that five activities decreased the subacromial space, participating in tendon lesions by friction on the undersurface of the acromion. As shoulder pathologies represent a paramount socioeconomic burden on the society and a cause of great distress to patients, the present findings are highly relevant. Indeed, sexual activities, if not performed with temperance, seem to be deleterious and may play a role in the dramatic surge in overall incidence rate in rotator cuff disease that has been recently reported [1].

The results of the present simulation seem to indicate that male or female can, in case of pain during intercourse, safely adopt a more passive attitude. Indeed, as expected, the watering can and the basset hound sexual positions required less stress on the rotator cuff and could be therefore considered as safer.

### Strengths and Limitations

This prospective study was the first to precisely analyze the impact of sexual positions on subacromial space height. The information helps the caregiver to advice patients suffering from rotator cuff pathologies. The findings are relevant and may explain the dramatic surge in overall incidence rate in rotator cuff disease [1]. Moreover, patient selection was strict with exclusion of all conditions (previous pathology or surgery, obsessive–compulsive disorder, etc.) that might affect the results. However, there were several limitations that warrant discussion. First, only two patients were tested due to the complexity of analysis and the sensitive nature of the experiment or delicate topic in which the subjects were asked to participate. This prevents us from verifying if similar patterns of subacromial impingements are observed in other individuals and correlating the results to patient-specific anatomy. Second, the body mass index of the two patients reflects reasonably healthy patients. Consequently, this study may underestimate the stress on rotator cuff generated by such activities in patients with higher body mass index. Third, the accuracy of the kinematics computation from motion capture data could be criticized. Gleno-humeral orientation and translation errors were respectively within 4° and 3 mm for each anatomical plane [9], which is acceptable for clinical use in the study of shoulder pathology. Fourth and last, we based our analysis of subacromial impingement on acromio-humeral distance [15]. Nevertheless, previous theories about acromio-humeral distance have been questioned. Indeed, it is unclear if the height of the subacromial space really plays a role, as it is now considered as a neo-articulation—the permanent contact between the humeral head and coraco-acromial arch during elevation of the arm being normal [16]. There is also growing evidence suggesting that distinct scapular morphologies and not simply subacromial impingement may accelerate the underlying degenerative process [17]. Despite these potential limitations, we do believe in the validity of the conclusions of this preliminary study.

## Conclusion

This study indicates that some sexual positions could potentially place the rotator cuff at risk. Such high-tech investigation shows promise in the areas of cause, intervention and education. The present findings may assist health professionals in providing them with preventive measures and is highly relevant for decision-making regarding health promoting initiatives.
